# Editorial: Embodying Tool Use: From Cognition to Neurorehabilitation

**DOI:** 10.3389/fnhum.2020.585670

**Published:** 2020-10-20

**Authors:** Giulia Galli, Yusuf Ozgur Cakmak, Jan Babič, Mariella Pazzaglia

**Affiliations:** ^1^Istituto di Ricovero e Cura a Carattere Scientifico (IRCCS) Fondazione Santa Lucia, Rome, Italy; ^2^Department of Anatomy, University of Otago, Dunedin, New Zealand; ^3^Brain Health Research Centre, Dunedin, New Zealand; ^4^Medical Technologies Centre of Research Excellence, Auckland, New Zealand; ^5^Centre for Health Systems and Technology, Dunedin, New Zealand; ^6^Laboratory for Neuromechanics and Biorobotics, Jožef Stefan Institute, Ljubljana, Slovenia; ^7^Department of Psychology, University of Rome “La Sapienza”, Rome, Italy

**Keywords:** embodiment, tool use, body representation, assistive devices, bodily illusions, robot, apraxia

Tool use is a ubiquitous, fundamental human characteristic that supports our ability to extend actions and thoughts beyond simple biological boundaries (Maravita and Iriki, 2004; Pazzaglia and Zantedeschi, 2016). The process of embodiment of tools may be defined as the sense that these non-corporeal objects extend bodily representations (Botvinick and Cohen, 1998) or become a “part of us” (Schettler et al.) In this framework, tool use consists of the mutual modification of the body, device, and world (i.e., other organisms, objects, the environment, or the self) (Pazzaglia and Molinari, 2016). Ownership of body parts or tools can be studied in patients after brain and spinal cord damage (Pazzaglia et al., 2018, 2020) as well as during experimental manipulations by using a virtual body and multisensory conflict (Botvinick and Cohen, 1998; Blanke et al., 2015). The scientific community has analyzed the sense of ownership (de Vignemont, 2011), the sense of agency (Pazzaglia and Galli, 2014), and the sense of self-location (Kilteni et al., 2012) to investigate the embodiment of corporeal objects and tools and their inherent bodily experience.

A substantial body of research on this Research Topic has focused on the developments and progress made in the embodiment of tools that extend the functionality of the body to support rehabilitation and to improve the capability for acting in the environment of patients with nervous system damage. Twenty-one novel publications have been collected that include 14 original research articles, 1 case report, 1 clinical trial, 3 reviews, 1 method, and 1 opinion.

Below is a brief overview of multiple original research contributions that focused on novel techniques, recognizing challenges, exploring new paradigms and methodologies, and integrating and expanding their theoretical reach. Taken together, these findings demonstrate the increasing attention and ongoing understanding with regard to how the body and the tool influence cognition.

Regarding the role of the body in clinical practice and especially in neurorehabilitation, Baumard and Osiurak have discussed the mechanisms underlying bodily experience as prerequisites for developing studies on tool incorporation. However, Weser and Proffitt noted that the subjective experience of the embodiment of a tool is quite different from that of experiencing body ownership. They argued that the body schema and the body image must work in harmony for one to experience a coherent sense of control over one's physical form as well as for identification with the physical form. However, it seems crucial to consider the different contributions of proprioception, sensory input from multiple modalities, body appearance, identity, position, tool function, proper grip, tool expertise, goals, and agency (Sun and Tang; Bruno et al.), as these factors cause different gradients of change in body representations.

Novel technologies are expected to open up new neurorehabilitation opportunities for the body. One of the reviews examined experimental and clinical studies that have explored the manipulation of an embodied virtual body in immersive virtual reality for experimental as well as clinical pain relief, suggesting the potential of using an embodied virtual body in immersive virtual reality (Matamala-Gomez et al.). The paper from Lancioni et al. presents an overview of recent technology-aided programs designed to help people with significant disabilities to engage in adaptive responses, functional activities, leisure, and communication and thus help them interact with their physical and social environment and improve their performance/achievement. Two contributions are innovative research approaches and neurorehabilitation techniques during childhood. These approaches involve the use of an augmented reality system for unveiling face topography in very young children (Miyazaki et al.) and the application of subthreshold vibrotactile noise stimulation to improve manual dexterity in a child with developmental coordination disorder (Nobusako et al.). The only study approaching a psychiatric condition, applied the rubber hand illusion in individuals with obsessive-compulsive disorder (OCD). This study suggests heightened malleability of body image in OCD and paves the way for a tolerable technique for the treatment of OCD (Jalal et al.).

Some of the studies have considered the applicability of virtual reality and robotic assistance in clinical populations with damage to the central nervous system. Borrego et al. determined and compared the sense of embodiment and presence elicited by a virtual environment under different perspectives and levels of immersion in individuals with stroke. Gandolfi et al. evaluated the effects of robot-assisted training on upper limb spasticity, function, muscle strength, and electromyographic muscle activity in chronic stroke patients treated with Botulinum toxin.

However, among the original research articles, a large group of contributions focused on promising interventions for more conventional rehabilitation following motor impairment after brain lesions (Borrego et al.; Pizzamiglio, Zhang, Kolasinski et al.; Gandolfi et al.; Spichala et al.; Pizzamiglio, Zhang, Duta et al.). Spichala and colleagues explored self-paced embodiable neurofeedback for post-stroke motor rehabilitation (Spichala et al.). Pizzamiglio et al. showed that patients with apraxia have difficulties in selecting elements of object-directed actions pertaining to habitual as well as goal-directed factors. Additionally, a few studies have further examined the neural underpinning of the action observation network (Pazzaglia and Galli, 2019) in relation to object use and effector independence through the application of voxel-based lesion-symptom mapping (Pizzamiglio et al.) and transcranial magnetic stimulation (Betti et al.).

Patients with brain lesions were not the only clinical population considered in this Research Topic. One of the contributions focused on the use of outcome measures to assess various areas of interest pertaining to lesions of the spinal cord (Scivoletto et al.). Surprisingly, this study revealed that the majority of the respondents did not evaluate the use of assistive technology. This result emphasizes the need for more thorough knowledge and use of outcome scales for improvement in the quality of assistive device evaluation (Galli and Pazzaglia, 2015). The theme of assistive device evaluation has also been considered in patients with transradial amputation (Cuberovic et al.). This study examined how passive learning of a home-used, neural-connected, and sensory-enabled prosthetic hand influenced the perception of artificial sensory feedback in the user. Participant interviews indicated that the naturalness of the experience and engagement with the prosthesis progressively increased, suggesting that artificial somatosensation may decrease prosthesis abandonment. From a clinical perspective, these results support the use of new rehabilitation perspectives that are necessary for successful integration of rapidly evolving technology into more effective rehabilitation practices aimed at restoring a sense of integrity in a damaged body.

The final theme that we identified in the contribution centers around innovative methodology, pioneering paradigms, and novel techniques. Using a multi-channel electroencephalogram (EEG) for automatic emotion recognition can help brain-inspired robots in reading people's interactive intentions and states (Xing et al.). This novel framework consists of a linear EEG mixing model and an emotion timing model. Building the linear EEG mixing model and decomposing the EEG source signals from the collected EEG signals improves the temporal classification of the EEG feature sequences, elected as the emotion classifier. Endo et al. used an adapted intentional binding effect paradigm to investigate whether the sense of agency can be used to measure the quality and experience of physical human-machine interaction schemes that allow the human operator and the collaborative machine to act as a “single entity.” One of the studies used functional near-infrared spectroscopy to measure the effects of moderate-intensity and high-intensity short-term aerobic exercise on prefrontal activity related to food images and recorded the subjective feelings of appetite in methamphetamine-dependent users (Wang et al.).

In conclusion, this Research Topic collects an impressive body of literature on “Embodying Tool Use.” Overall, the contributions extend and enrich the previous multidisciplinary approach and translational applications. However, despite the significant progress made in our understanding and the real-world relevance, there are boundless directions, endless possibilities, and exciting challenges yet to be explored in future research.

## Author Contributions

MP planned the topic. MP and GG edited the most of papers included in the topic. YC and JB edited some papers included in the topic. GG drafted the editorial which all authors reviewed and approved. All authors contributed to the article and approved the submitted version.

## Conflict of Interest

The authors declare that the research was conducted in the absence of any commercial or financial relationships that could be construed as a potential conflict of interest.
